# Intravenous iron supplementation does not increase infectious disease risk in hemodialysis patients: a nationwide cohort-based case-crossover study

**DOI:** 10.1186/s12882-019-1495-7

**Published:** 2019-08-22

**Authors:** Chieh-Li Yen, Yu-Sheng Lin, Yueh-An Lu, Hsin-Fu Lee, Cheng-Chia Lee, Ying-Chang Tung, George Kuo, Lung-Sheng Wu, Ya-Chung Tian, Pao-Hsien Chu

**Affiliations:** 1 Division of Nephrology, Department of Internal Medicine, Chang Gung Memorial Hospital, Chang Gung University, College of Medicine, Taipei, Taiwan; 2 Division of Cardiology, Department of Internal Medicine, Chang Gung Memorial Hospital, Chang Gung University College of Medicine, 199 Tun-Hwa North Road, Taipei, Taiwan

**Keywords:** Iron, Intravenous, Infection, Hemodialysis, ESRD

## Abstract

**Background:**

Studies have reported conflicting findings on the infection risk posed by intravenous iron supplementation among hemodialysis (HD) patients. We used a novel study design to assess associations between intravenous iron and infectious diseases.

**Methods:**

Patients initiating HD between 1998 and 2008 were extracted from Taiwan’s National Health Insurance Research Database. Their first infectious disease in the period between 1.5 years after dialysis initiation and 2010 was identified and defined as the index date. Through the case-crossover design, the odds of exposure to intravenous iron within the 1-month period immediately preceding the index date (i.e., the case period) were compared with iron exposure in three different matched control periods for the same enrollee, thus possibly reducing some unmeasured confounders.

**Results:**

A total of 1410 patients who met our enrollment criteria were extracted from incident HD patients. The odds of intravenous iron exposure during the case period versus total control periods exhibited no significant difference (odds ratio: 1.000, 95% confidence interval: 0.75–1.33). In subgroup analyses, this association remained nonsignificant across patients with diabetes mellitus, heart failure, chronic lung disease, venous catheter for HD, and higher iron load.

**Conclusions:**

We found that intravenous iron supplementation did not increase short-term infection risk among HD patients.

**Electronic supplementary material:**

The online version of this article (10.1186/s12882-019-1495-7) contains supplementary material, which is available to authorized users.

## Background

Anemia represents a major health problem among patients receiving maintenance hemodialysis (HD) and was reported to be associated with hospitalization and death [1]. Treatment of renal failure–related anemia is mainly based on erythropoiesis-stimulating agents (ESAs), but the hemoglobin level is occasionally difficult to achieve or maintain under ESA-only treatment. Iron deficiency due to frequent blood loss and diminished iron absorption in HD patients is a major contributing factor to poor ESA response [2, 3]. Many studies [4–6] have reported intravenous iron replacement to reduce the required ESA dose and increase hemoglobin levels.

With the extensive use of intravenous iron replacement in HD patients, several researchers have raised the concern that excessive iron load may increase the risk of adverse cardiovascular events [7] and infectious diseases [8, 9]. Regarding the association between intravenous iron therapy and infectious disease, which is the main focus of this study, in vitro studies have reported some iron-associated negative effects, including promoting bacterial growth [10] and impairing both innate [11, 12] and adaptive [13, 14] immune response. These immune dysfunctions may be more severe in HD patients, who receive numerous needle punctures during dialysis sessions, thus providing entry sites for bacteria. Although intravenous iron theoretically increases the risk of infectious disease among HD patients, in vivo studies have reported conflicting findings. Some studies demonstrated that intravenous iron supplementation increased the risk of infection and mortality [15, 16], but others found that there was no significant association between intravenous iron and all-cause mortality or infection-associated mortality [17, 18]. These inconsistent findings are partially attributable to the complexity of HD, which render it difficult to prevent residual confounders that can interfere with the final research results. According to our review of the literature, the following possible residual confounders have been mentioned in observational cohort studies: 1. dissimilar treatment strategies and hemoglobin targets at different dialysis centers may affect outcomes; 2. a history of infectious disease can influence the decision to initiate intravenous iron therapy and result in uneven distribution of patients; and 3. differences of nutritional status, and dietary habit across HD patients, although crucial to iron deficiency, are difficult to analyze in observational studies.

The limited and inconsistent evidence published thus far is insufficient for physicians to weigh the possible benefits and risks when considering intravenous iron treatment. By focusing on incident HD patients in a nationwide cohort and using a case-crossover design, we conducted this study with the aim of limiting possible confounders and providing more reliable information to elucidate the association between intravenous iron supplementation and bacteria-related infectious diseases in HD patients.

## Methods

### Data source

We performed this study by using the Registry for Catastrophic Illness Patient Database (RCIPD), which forms part of Taiwan’s National Health Insurance Research Database (NHIRD). Taiwan has a nationwide, single-payer, compulsory health care program covering nearly 23.37 million people, or 99.9% of its population in 2014. The NHIRD contains detailed health care information on insured people, including outpatient visits, hospital admissions, drug prescriptions, procedures, and beneficiaries. Disease diagnoses in the NHIRD are made in accordance with the International Classification of Diseases, 9th Revision, Clinical Modification (ICD-9-CM). Even though laboratory data and examination reports are not available in the database, the population-wide comprehensiveness of NHIRD is a strength of the study.

The RCIPD, a subset of the NHIRD, contains claims data from patients with certain chronic and severe conditions, including end-stage renal disease (ESRD), certain autoimmune diseases, and malignancies, who thus qualify for a catastrophic illness certificate (relieving insured patients of copayments). To protect personal privacy, any data that could identify a specific patient or health care provider in the NHIRD and RCIPD are scrambled before being released. Therefore, this study qualified for a waiver of consent, which was approved by Chang Gung Medical Foundation’s Institutional Review Board (approval number: 201801731B1).

### Study design: case-crossover study

The case-crossover study design enrolls only patients with outcome events. For each enrolled patient, possible exposure to the risk factor of interest during the period immediately preceding the outcome event (i.e., the case period) is compared with exposure during several periods without the outcome event (i.e., control periods). Through the use of patients as their own control, this study design can reduce selection bias and unmeasured confounders. It is particularly suitable for investigating the association between risks of acute outcomes and the short-term effect of intermittent exposure [19, 20]. In Taiwan, HD patients usually receive an intravenous 100 mg-iron once or twice a week for 1 to 3 months to achieve a 1-g supplementation or 100 mg-iron once every two weeks to achieve a 500-mg supplementation, if required according to the attending physician’s evaluation. However, a long-term maintenance dosing of iron supplementation is seldom used. Considering this strategy of intermittent iron therapy among Taiwanese HD patients, a case-crossover study design was considered suitable for assessing whether intravenous iron treatment increases the risk of infectious disease.

### Patient selection

**Fig. 1:**
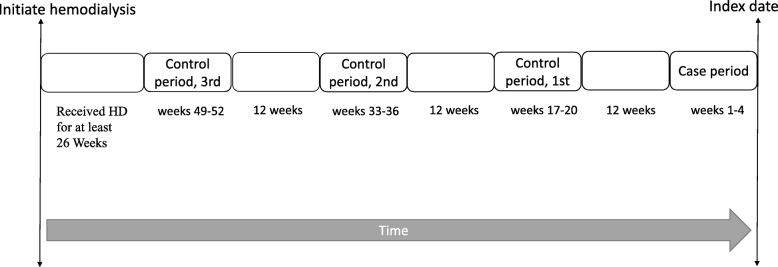
Time frame of case and control periods**.** This case cross-over design involved only cases who experienced infectious disease needed intravenous antibiotics treatment, and each case served as his/her own control. The index date defined as the first date of intravenous antibiotics after commencing hemodialysis

As shown in Fig. 1, adult patients with new onset of ESRD who initiated long-term HD between 1998 and 2008 were identified in the RCIPD. Those whose treatment changed to peritoneal dialysis or who received a renal transplant were excluded from this study to avoid the change of renal replacement therapy (from HD to renal transplant, from HD to PD or vice versa) during the observational period, which may further bias the results. Patients experiencing their first infectious disease after commencing HD were identified. The definition of “infectious disease” for inpatients was ascertained by identifying hospitalizations with first three discharge diagnosis of selected ICD-9-CM codes along with more than two days of intravenous antibiotics treatment. For outpatients, “infectious disease” was defined by more than two claims of diagnosis of infectious disease within one month along with more than two consecutive days of intravenous antibiotics treatment in outpatient or HD center visits. All ICD-9-CM codes used in the study to identify an infectious disease were listed in Additional file 1: Table S1. The first date of antibiotic treatment served as the index date. Because the case-crossover design only permitted enrollment of patients with outcome events, those without any infectious disease between their first HD date and 2010 were excluded. In addition, patients with any infectious disease within 1.5 years (78 weeks) after HD initiation were excluded. The reason to exclude these patients will be explained in detail in the next section “case and control periods”.

### Case and control periods

**Fig. 2:**
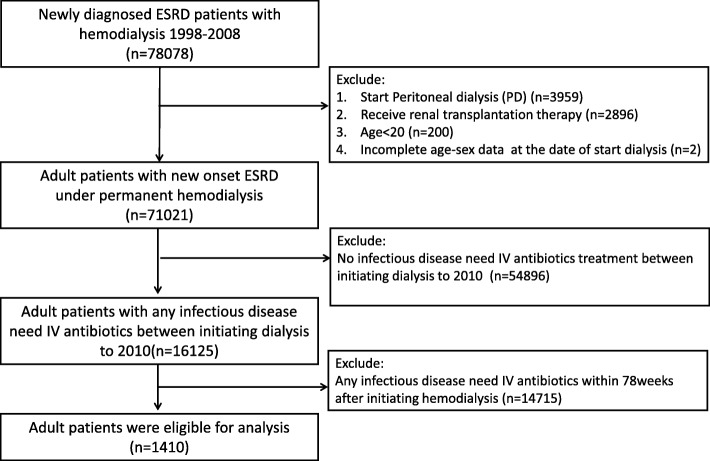
Inclusion of study patients

Figure 2 illustrates the time frame of case and control periods in this study.

A period of 4 weeks immediately preceding the index date was defined as the “case period,” given that the incubation periods of *Escherichia coli*, *Staphylococcus aureus*, and *Streptococcus pneumoniae*, the most common infectious pathogens among HD patients [21], are 3–8 days, 4–10 days, and 1–3 days, respectively (data from WHO website). In addition, in vitro study [12] has proved that intravenous iron can impair the bactericidal ability of polymorphonuclear leukocytes within a 24-h period. Thus, a 4-week period represents a suitable “at risk” interval for infectious disease. Each case period was matched to three 4-week control periods. The case and control periods were separated from each other by 12 weeks to avoid carryover effects, given that, according to our database, the mean treatment duration among HD patients who require intravenous iron supplementation is 3.09 months per year (Table 1), and usually divided into several treatment courses. Because patients mostly experienced many clinical changes during the first several months after commencing dialysis, such as transferring from medical center to local dialysis room with possibly different iron supplementation policy and changing dialysis access from venous catheter to fistula., which might interfere the results. We maintained a time interval of at least half a year (26 weeks) between the first HD date and the start of the observation period to reduce these confounders. The method was also adopted by a previous high quality research (a 9-month interval in their design) [15]. In addition, as patients seldom receive regular intravenous iron supplementation before commencing dialysis, the odds of exposure to iron supplementation may be biased if part of observational periods preceded the initiation of dialysis. Consequently, patients with their first infection within 78 weeks after initiation of HD were excluded to ensure all enrollee’s observational periods at least 6 months after starting HD.

**Table 1:** Characteristics of the all study patients.(*n* = 1410)

Characteristics	Number of Patients (%)
Age, year	
18–35	25 (1.8)
35–45	64 (4.5)
45–55	223 (15.8)
55–65	328 (23.3)
65–75	434 (30.8)
> 75	336 (23.8)
Mean Age (standard deviation)	65.09 (12.65)
Female	780 (55.3%)
Iron dosage during treatment	
0 mg/month	1127 (79.9)
0-300 mg/month	162 (11.5)
300-400 mg/month	31 (2.2)
> 400 mg/month	90 (6.4)
Mean Iron treatment duration, months/year (standard deviation)^a^	3.09 (2.17)
Permanent venous catheter on index day	49 (3.5)
Comorbidities within 2 years before index date	
Hypertension	1142 (81.0)
Ischemic heart disease	690 (48.9)
Stroke	278 (19.7)
Diabetes mellitus	849 (60.2)
Chronic pulmonary disease	317 (22.5)
Heart failure	437 (31.0)
Liver cirrhosis	102 (7.2)
Connective tissue disease	215 (15.2)
Malignancy	244 (17.3)
Polycystic kidney disease	13 (0.9)

^a^patients without intravenous iron treatment were excluded

### Identification of intravenous Iron exposure

Intravenous iron exposure was defined as any registered claim of intravenous iron dextran, iron sucrose, and sodium ferric gluconate, which are the only three intravenous iron products available in Taiwan, during case or control periods.

### Study outcomes and statistical analysis

The distribution of age, sex, comorbidities, access for HD on the index date, and mean monthly dose of intravenous iron supplementation during treatment are presented. On the basis of ICD-9-CM diagnostic codes, comorbidities were identified from more than two outpatient claims or any inpatient claim within 2 years preceding the index date. The diagnostic codes for comorbidities have been validated in other NHIRD-based studies [22, 23]. For analysis in this case-crossover study, we used conditional logistic regression to estimate the odds ratio for the likelihood of intravenous iron exposure during the case period, compared with its matched control periods. We conducted a test for interaction, using logistic regression model, to evaluate for any significant subgroup difference. A *p*-value of < 0.05 was considered statistically significant. All statistical analyses were performed using SAS version 9.4 (SAS Institute, Cary, NC, USA).

## Results

### Patient characteristics

A total of 1410 adult patients who contracted their first infectious disease needed continuous intravenous antibiotics treatment after commencing HD met our enrollment criteria. The main patient characteristics are listed in Table 1. The mean age was 65.09 ± 12.65 years, and 55.3% of the patients were female. Among the enrollees, 20.1% received intravenous iron supplementation, and 8.6% received more than 300 mg of iron per month during treatment. The mean duration of iron treatment among patients received intravenous iron supplementation is 3.09 months per year. Regarding HD type, only 3.5% of the patients used an venous catheter, and the rest used an arteriovenous fistula or graft. The prevalence rate of co-morbidities reported in this study is similar to them of previous NHIRD-based studies using whole cohort of ESRD patients [24, 25].

### Association between Iron exposure and infection

Table 2 presents the distribution of iron exposure, indicating the odds ratio of iron exposure between case and control periods. Of the 1410 HD patients in this study, 4.8% were exposed to intravenous iron treatment in the case period, 4.4% in control period 1, 4.5% in control period 2, and 5.3% in control period 3. The odds of intravenous iron exposure exhibited no significant difference between the case period and control period 1 (odds ratio (OR) 1.08, 95% confidence interval (CI) 0.76–1.54), control period 2 (OR 1.05, 95% CI 0.74–1.49), control period 3 (OR 0.89, 95% CI 0.63–1.29), and total control periods (OR 1.000, 95% CI 0.75–1.33). Overall, the proportion of intravenous iron exposure during the case period preceding infectious disease was not significantly higher than that during the matched control periods.

**Table 2:** Number of patients and the odds ratio of iron exposures between case and control periods (*n* = 1410)

	Exposure to Iron (%)		Case period versus Control period OR (95% CI)	*P*-value
Case period		67 (4.8%)		
Control period 1		62 (4.4%)	1.08 (0.76,1.54)	0.652
Control period 2		64 (4.5%)	1.05 (0.74,1.49)	0.788
Control period 3		75 (5.3%)	0.89 (0.63,1.25)	0.491
Control period 1 + 2 + 3		201 (4.8%)^a^	1.00 (0.75,1.32)	1.000

^a^The total number of subjects for control period 1 + 2 + 3 is 4230

### Subgroup analysis

Although intravenous iron supplementation appeared not to represent a risk of infectious disease in overall patients, we also wished to investigate whether this finding remained consistent in different clinical situations. Therefore, we performed subgroup analyses for the odds of iron exposure between case and control periods among patients under the following conditions: 1. comorbidities of diabetes mellitus (OR 1.008, 95% CI 0.710–1.432), heart failure (OR 1.070, 95% CI 0.680–1.684), and chronic lung disease (OR 1.141, 95% CI 0.643–2.024); 2. venous catheter for HD (OR 0.000, 95% CI 0.000–999.99); and 3. higher intravenous iron load (> 300 mg/month) during treatment (OR 0.839 95% CI 0.508–1.386) (Table 3). All of these conditions proved to carry a high risk of infectious disease in HD patients [26, 27]. However, according to the subgroup analysis results, the proportion of intravenous iron exposure was not significantly different during the case and control periods for any subgroup in this study. Therefore, even in patients with high prevalence of infectious disease, intravenous iron supplementation didn’t increase the risk of severe infection.

**Table 3:** Subgroup Analysis

	Iron treatment in case period(%)	Iron treatment in control period 1 + 2 + 3(%)	Odds ratio (95% CI)	*P*-value	*P*-value for interaction
Heart failure					0.710
Yes	27 (6.2%)	76 (5.8%)	1.070 (0.680, 1.684)	0.769	
No	40 (4.1%)	125 (4.3%)	0.958 (0.666, 1.379)	0.818	
Chronic lung disease					0.607
Yes	17 (5.4%)	45 (4.7%)	1.141 (0.643, 2.024)	0.652	
No	50 (4.6%)	156 (4.8%)	0.960 (0.693, 1.330)	0.805	
Diabetes Mellitus					0.940
Yes	44 (5.2%)	131 (5.1%)	1.008 (0.710, 1.432)	0.964	
No	23 (4.1%)	70 (4.2%)	0.985 (0.609, 1.594)	0.951	
Venous catheter for HD					0.976
Yes	0 (0%)	7 (4.8%)	0.000 (< 0.001, > 99.99)	0.952	
No	67 (4.9%)	194 (4.8%)	1.038 (0.781, 1.380)	0.796	
Iron > 300 mg/month					0.391
Yes	25 (21%)	86 (23%)	0.839 (0.508, 1.386)	0.493	
No	42 (3.3%)	115 (3.0%)	1.099 (0.767, 1.574)	0.607	

## Discussion

Using the NHIRD, we conducted a retrospective case-crossover study to elucidate the association between intravenous iron supplementation and bacterial infectious disease among patients under permanent HD. Because of the diversity and complexity of clinical situations among such patients, to simplify the study population and to avoid the interdependence of repeated measures, we focused on to patients’ first infectious disease after initiating HD. Under this enrollment criterion, we found that intravenous iron supplementation was not associated with increased risk of infectious disease requiring intravenous antibiotic treatment. This result was consistent across different clinical conditions, namely HD via venous catheter; higher monthly iron dosage; and comorbidities of diabetes mellitus, chronic pulmonary disease, and heart failure.

Although previous in vitro or animal studies have demonstrated that a large iron dosage can induce immune dysfunction [12, 28] and support bacterial growth [10], the evidence from clinical studies is contradictory. Among earlier large cohort studies, which have usually focused on intravenous iron and long-term infection risk, two have found a large iron dose harmful [29, 30], one found no association [17], and another found that it could be beneficial only in patients with severe anemia [16]. By contrast, more recent studies have mainly focused on the short-term effect of intravenous iron supplementation. One observational study [15] of 117,050 HD patients examined whether 1-month intravenous iron exposure increases infectious disease risk in subsequent 3-month periods, and Brookhart et al. found a higher risk of infection-related hospitalization among patients receiving iron bolus treatment or a higher iron dosage. By contrast, one cohort study [18] using data from the US Renal Data System demonstrated that intravenous iron supplementation during infectious disease associated with hospitalization did not increase mortality risk during hospitalization. Another large cohort study [31] of incidental HD at Dialysis Clinic Inc. facilities showed no association between 1-, 2-, or 6-month cumulative intravenous iron doses and infection or mortality within the following month. We believe that these inconsistent findings may be partially attributed to residual confounders, because HD patients represent a variety of clinical situations, such as disability, malnutrition, and immunodeficiency, which were difficult to evaluate with database. Moreover, the interaction of these situations and comorbidities diseases, such as diabetes mellitus, heart failure, autoimmune diseases, or polycystic kidney disease further complicates their clinical conditions. The complexity and diversity of HD patients render certain unmeasured confounders inevitable, thus further possibly hindering the research outcomes.

Our study is novel in its use of a case-crossover design to evaluate the association between iron and infection, regarding patients as their own control to reduce plausible residual confounders such as genetic and ethnic differences, different treatment strategies among HD institutions, nutrition status, or patient performance. In addition, we defined the first infectious disease date after HD initiation as the index date and retrospectively observed intravenous iron use during the year preceding the index date. The enrollment criteria thus ensured that patients in our study were free of severe infectious diseases during the time between dialysis initiation and the index date. This design could prevent the uneven distribution of enrollees. For example, a prior severe infectious disease could affect a decision regarding further intravenous iron supplementation and thus produce an unmeasured confounder. Meanwhile, confining to first infection help avoid the interdependence of repeated measures since patients who get infection are probably more vulnerable to further infections. Therefore, we found no association between intravenous iron supplementation and short-term bacteria-associated infectious disease in the overall cohort.

Some studies [26, 27] have indicated that certain comorbidities could increase the risk of infectious disease among HD patients, namely heart failure, chronic pulmonary disease, and diabetes mellitus. One study also reported that venous catheter use could increase the risk of sepsis by providing an entry site for bacteria [32]. With regard to these high-risk populations, we demonstrated that intravenous iron treatment did not increase the infection risk across subgroups. Dosing effect is another topic of great interest, with studies reporting conflicting results [15, 31]. Similarly, we found no trend of increased infectious disease risk among patients receiving higher iron doses (> 300 mg/month). Notably, the number of patients of this study is relatively insufficient to achieve a satisfactory power in subgroup analysis, especially in analysis of dialysis accesses, and thus made the results uncertain. Further studies specific for different subgroups are warranted to validate our findings.

Pivotal study [33], a recent multicenter, open-label trial, randomly assigned patients under hemodialysis to receive either high-dose intravenous iron supplementation (400 mg/month) or low-dose intravenous iron supplementation (0-400 mg/month). In this study, Iain C. Macdougall, et al. demonstrated the higher monthly dose of intravenous iron supplementation did not increase the infection rate to the low-dose iron supplementation. Compared to Pivotal study, which offered evidence on long-term safety of higher dose iron regimen, our research focused on short-term effect and demonstrated intravenous iron supplementation did not increase short-term infection rate.

Some limitations of this study should be acknowledged. First, laboratory data, including serum albumin, ferritin, transferrin, and hemoglobin, were not available to us in the NHIRD. Besides, the latest data of NHIRD is not available in this study. Thus, temporary differences in anemia management might influence our result and further studies are warranted. Second, a case-crossover study design is only useful in analyzing short-term effects. The relationship between long-term cumulative iron exposure and the risk of infectious disease, which was possibly more important for clinician to know, was thus beyond the scope of this study. Third, our research focused only on infectious diseases requiring intravenous antibiotic treatment. Therefore, certain chronic infectious diseases commonly treated using oral antibiotics, such as tuberculosis, were not covered by this study. We intend to design further research to elucidate the role of intravenous iron in such populations. Fourth, because evaluating the association between iron exposure and infectious disease by means of a case-crossover study represents a novel approach, selection of appropriate case and control periods can only be based empirically on other studies’ designs and evidence from in vitro studies. Fifth, we exclude patients with an infectious disease within 1.5 years after initiation of HD to avoid some confounding. However, the exclusion criteria yield those patients who experienced an early infectious disease after starting HD unable to analyze in this study.

## Conclusions

In conclusion, by reducing certain residual confounders poorly eliminated in a general study design, we provide evidence to support the short-term safety of intravenous iron supplementation in HD patients at risk of infectious diseases. However, since one observational study is impossible to remove all potential confounders regardless of the research design, randomized controlled trials are the only scientifically robust means of ascertaining the truth. Additional well-designed, large-scale randomized controlled trials are warranted for further validation.

## Additional file

Additional file 1: **Table S1.** Description of data: ICD-9-CM codes used to identify a meaningful infection. (DOCX 12 kb)

## Data Availability

All data generated or analysed during this study are included in this published article.

## Abbreviations

ESAs: Erythropoiesis-stimulating agents
ESRD: End-stage renal disease
HD: Hemodialysis
NHIRD: Taiwan’s National Health Insurance Research Database
RCIPD: Registry for Catastrophic Illness Patient Database
