# Supplementary material for: Intravenous iron supplementation does not increase infectious disease risk in hemodialysis patients: a nationwide cohort-based case-crossover study
Source: BMC Nephrol. 2019 Aug 22;20:327. doi: 10.1186/s12882-019-1495-7 (PMC6704706; doi:10.1186/s12882-019-1495-7)
Supplement: Supplementary file 1 — Table S1. Description of data: ICD-9-CM codes used to identify a meaningful infection. (DOCX 12 kb) [file 12882_2019_1495_MOESM1_ESM.docx]

**Additional file 1: Table S1. ICD-9-CM codes used to identify a meaningful infection**

| **Infectious disease** | **ICD-9-CM codes** |
| --- | --- |
| Sepsis | 038, 995.91, 995.92, 020.2, 785.52, 790.7 |
| Pneumonia | 481-486 (exclude 484) |
| Empyema | 510 |
| Cellulitis | 681, 682 |
| Necrotizing fasciitis | 728.86 |
| Urinary tract infection | 590, 595.0, 599.0 |
| Biliary tract infection | 576.1, 575.0, 574.00 |
| Brain abscess | 324 |
| Liver abscess | 572.0 |
| Perianal abscess | 566 |
| Bacterial meningitis | 320 |
| Septic arthritis | 711 |
| Infection of catheter, device, implant, and graft | 996.6, 999.3 |
| Peritoneal and retroperitoneal infection | 567.0, 567.1, 567.2, 567.3 |
| Osteomyelitis | 730.3, 730.8, 730.9 |
| Infective endocarditis | 421 |
